# Preparation and Optimization of Silver Nanoparticle-Loaded Dendritic Fibrous Membranes for High-Efficiency Antibacterial Activity and Air Filtration

**DOI:** 10.3390/mi17050614

**Published:** 2026-05-16

**Authors:** Yang Huang, Bofeng Li, Zhongyi Yu, Xianruo Du, Ruixin Chen, Xiang Wang, Jiaxin Jiang, Gaofeng Zheng, Huatan Chen

**Affiliations:** 1 School of Mechanical and Automotive Engineering, Xiamen University of Technology, Xiamen 361024, China; 2522021023@stu.xmut.edu.cn (Y.H.); 2522021030@stu.xmut.edu.cn (B.L.); 18460089028@163.com (Z.Y.); wx@xmut.edu.cn (X.W.); jjx@xmut.edu.cn (J.J.); 2 College of Physical Science and Technology, Xiamen University, Xiamen 361005, China; duxianruo@stu.xmu.edu.cn; 3 Pen-Tung Sah Institute of Micro-Nano Science and Technology, Xiamen University, Xiamen 361102, China; chenruixin@stu.xmu.edu.cn (R.C.); zheng_gf@xmu.edu.cn (G.Z.)

**Keywords:** silver nanoparticles, antibacterial activity, electrospinning, air filtration, dendritic fibers

## Abstract

Metal nanoparticles are widely used in fibrous membrane materials due to their excellent antibacterial properties. However, metal nanoparticle-loaded fibrous membranes often face the trade-off between antibacterial performance and filtration efficiency. To address this issue, silver nanoparticle-loaded dendritic fibrous membranes were prepared via electrospinning technology in this study, and the dual optimization of antibacterial and filtration performance was achieved by adjusting the silver loading amount and fiber morphology. The results showed that the prepared silver nanoparticle-loaded PVDF dendritic fibrous membrane exhibited an outstanding air filtration performance with a filtration efficiency of 99.87% for 0.3 µm particulate matter, a pressure drop of 87.4 Pa, and a quality factor (QF) of 0.076 Pa^−1^. In addition, the membrane presented excellent antibacterial activity with inhibition rates of 99.9% and 99.8% against *Escherichia coli* and *Staphylococcus aureus*, respectively. This study provides a new insight into resolving the trade-off between air filtration and antibacterial performance of metal nanoparticle-loaded fibrous membranes and offers an important reference for applications in related fields.

## 1. Introduction

Air pollution has become a major global public health challenge. Especially with accelerated urbanization, air pollutants such as fine particulate matter, harmful gases and pathogenic microorganisms pose a serious threat to human health [1,2,3]. Nanofiber membranes have become the most promising air filtration materials in recent years, and related preparation technologies, performance optimization strategies and application scenarios have been systematically reviewed in top academic journals [4,5]. Although traditional air filtration materials can effectively filter large particulate pollutants, they show low efficiency in removing fine particulates smaller than 0.3 µm and usually lack antibacterial functions [6,7,8]. This not only leads to the risk of reduced filtration performance during long-term use but also makes these materials prone to becoming a breeding ground for bacteria [9,10]. Therefore, the development of high-efficiency, long-lasting air filtration materials with antibacterial functions has become an urgent need to solve this problem [11,12].

At present, metal nanoparticles are widely used in fibrous membrane materials due to their low minimum inhibitory concentration (MIC) and excellent antibacterial properties [13]. Copper-based nanoparticles (e.g., Cu, CuO, Cu_2_O) have been applied in fibrous membranes for inhibiting bacterial growth and antivirus, showing good antibacterial effects [14]. Zinc and iron metal nanoparticles significantly enhance the antibacterial properties of fibrous membranes through mechanisms such as releasing metal ions and generating reactive oxygen species (ROS) [15]. Among metals, silver nanoparticles are the most commonly used antibacterial materials due to their remarkable antibacterial activity and low MIC. Silver exhibits excellent performance in inhibiting bacteria, viruses and fungi, and can achieve significant antibacterial effects at low loading amounts, thus becoming one of the most ideal antibacterial materials at present [16,17,18,19]. A large number of studies have confirmed that loading silver nanoparticles into electrospun nanofibrous membranes can simultaneously achieve efficient antibacterial function and optimized air filtration performance, which has become a mainstream research direction in this field [20,21,22].

Despite the excellent antibacterial performance of metal nanoparticles, their loading in fibrous membranes still faces the challenge of structural optimization. The introduction of metal nanoparticles can significantly improve the antibacterial performance of membranes, but it often causes structural changes in fibrous membranes, thereby affecting their filtration efficiency [23]. Specifically, with the increase in metal nanoparticle loading, the pore size distribution and air permeability of fibrous membranes may be affected, leading to the decline in filtration performance [24]. Existing studies focus on improving the antibacterial effect of metal nanoparticles, but there are still deficiencies in balancing antibacterial performance and filtration efficiency. Especially in the filtration of fine particulates, metal nanoparticle-loaded membranes face challenges in achieving both ideal filtration effect and low pressure drop simultaneously.

To solve this problem, the electrospinning process has become an ideal preparation method due to its ability to precisely control the diameter, morphology and pore structure of fibers [25,26,27,28]. Electrospinning technology can prepare fibrous membranes with highly tunable structures, which can effectively optimize the pore size distribution and air permeability of fibrous membranes, thereby improving filtration efficiency while maintaining a low pressure drop [29]. In addition, factors such as solution conductivity and viscosity during the electrospinning process can further regulate the loading amount and distribution of metal nanoparticles to achieve the balance between antibacterial and filtration performance [30,31].

Aiming to address the current problem of balancing antibacterial and filtration performance of metal nanoparticle-loaded fibrous membranes, this study proposed a new solution. Silver nanoparticle-loaded polyvinylidene fluoride (PVDF) fibrous membranes were prepared by electrospinning technology, and the pore size distribution and air permeability of the membranes were optimized by adjusting the silver loading amount and fiber morphology. These structural adjustments effectively improved the filtration efficiency of the membranes and maintained a low pressure drop, thus enhancing the antibacterial performance while ensuring high-efficiency air filtration. In addition, the uniform loading of silver nanoparticles was ensured and their antibacterial effect was maximized by controlling the distribution and morphology of silver nanoparticles. Through such structural and material optimization, this study successfully solved the trade-off between air filtration and antibacterial performance of metal nanoparticle-loaded fibrous membranes, and provided a new theoretical basis and technical approach for the development of high-efficiency air filtration materials. Notably, the multifunctional dendritic fibrous membrane prepared in this work can be integrated as a core functional component into micro/nano air purification devices, wearable respiratory protection microsystems, and microfluidic chip sterilization modules, thereby expanding its application potential in the field of micro/nano device engineering.

## 2. Materials and Methods

### 2.1. Reagents and Materials

Polyvinylidene fluoride (PVDF) was purchased from Shanghai Yuanye Bio-Technology Co., Ltd. (Shanghai, China). Silver nitrate (AgNO_3_, purity > 99%) was obtained from Beijing InnoChem Science & Technology Co., Ltd. (Beijing, China). Polyvinylpyrrolidone (PVP) was purchased from Shanghai Jizhi Biochemical Technology Co., Ltd. (Shanghai, China). Isopropanolamine (MIPA) was obtained from Shanghai Amoer Biotech Co., Ltd. (Shanghai, China). N,N-Dimethylformamide (DMF) was purchased from Beijing InnoChem Science & Technology Co., Ltd. (Beijing, China). Acetone (AC) was obtained from Shanghai Chuangsai Technology Co., Ltd. (Shanghai, China). Sodium chloride (NaCl) (purity ≥ 99.5%) was obtained from China National Pharmaceutical Group Chemical Reagents Co., Ltd. (Shanghai, China). The rationale for selecting these core materials is as follows:

PVDF was chosen as the polymer matrix due to its excellent mechanical properties, weather resistance, spinnability, and piezoelectric characteristics that can enhance electrostatic adsorption during filtration. Compared with PLA and PAN, PVDF exhibits superior hydrophobic stability and long-term service performance, making it a widely used matrix for commercial air filtration membranes. AgNO_3_ was selected as the silver source because of its high solubility in DMF/AC mixed solvent, which allows in situ reduction during electrospinning. Unlike pre-synthesized silver nanopowders, AgNO_3_ avoids particle agglomeration and enables uniform silver distribution on the fiber surface. PVP and MIPA were included as chelating/reducing agents, as they are commonly used in electrospinning systems to regulate Ag^+^ reduction and solution properties, with distinct effects on rheology and fiber morphology.

### 2.2. Preparation of Electrospun Membranes

Firstly, PVDF was dissolved in a mixed solvent of DMF and AC (volume ratio 8:2) to prepare a PVDF solution with a concentration of 9% (*w*/*v*, g/100 mL). Then, AgNO_3_ and PVP were added to the solution at different ratios, and an appropriate amount of MIPA was added as a complexing agent. To investigate the effects of different components, six groups of solutions were prepared, corresponding to samples A, B, C, D, E and F, respectively. To investigate the effects of different components, six groups of solutions were prepared, and the corresponding nanofibrous membranes were named according to their components and silver loading amount. The full nomenclature and solution formulations are shown in the Table 1—Pure PVDF membrane: blank PVDF membrane without any additives (original sample A)—PVDF/AgNO_3_ membrane: PVDF membrane with only AgNO_3_ added (original sample B)—PVDF/AgNO_3_/PVP-L membrane: PVDF membrane with low loading of AgNO_3_ and PVP added (original sample C)—PVDF/AgNO_3_/PVP-H membrane: PVDF membrane with high loading of AgNO_3_ and PVP added (original sample D)—PVDF/AgNO_3_/MIPA-L membrane: PVDF membrane with low loading of AgNO_3_ and MIPA added (original sample E)—PVDF/AgNO_3_/MIPA-H membrane: PVDF membrane with high loading of AgNO_3_ and MIPA added (original sample F). The solution formulations are shown in the table below (all values in the table represent the absolute mass of pure raw materials; the PVDF column refers to the mass of pure PVDF powder, not the mass of pre-prepared PVDF solution):

This experiment adopts a single-factor controlled variable design, with sample A as the blank substrate control. The core variables are the loading amount of the silver precursor and the type of complexing agent; among them, sample B serves as a methodological validation control group, used to verify the role of the complexing agent in the construction of the target structure, and is not included in the performance analysis of the core experimental group.

All solutions were stirred for 12 h to ensure complete dissolution and form homogeneous and precipitate-free solutions. Subsequently, the prepared solutions were electrospun using an electrospinning machine (Foshan Lepton Precision Measurement and Control Technology Co., Ltd., Foshan, China). A horizontal electrospinning setup was employed, where the spinneret and the grounded roller collector were positioned at the same horizontal height. The solution was loaded into a 21G needle. The distance between the spinneret and the collector was 15 cm, the applied voltage was 22 kV, and the liquid supply rate was 300 µL/h. The temperature was maintained at 25 ± 2 °C and the relative humidity at 50 ± 3%. The electrospun nanofibers were deposited on a grounded roller covered with PP non-woven fabric, and then dried in a vacuum oven at 60 °C for 12 h to remove residual solvents, yielding nanofibrous membranes. Six types of nanofibrous membranes were obtained from the six groups of electrospinning solutions, corresponding to samples A, B, C, D, E and F, among which B, C, D, E and F were silver nanoparticle-loaded fibrous membranes.

### 2.3. Determination of Solution Properties

To investigate the solution properties, the viscosity and conductivity of the solutions were measured and compared using a viscometer (NDJ-1, Shanghai Hengping Scientific Instrument Co., Ltd., Shanghai, China) and a conductivity meter (DDS-307A, Shanghai INESA Scientific Instrument Co., Ltd., Shanghai, China), respectively.

### 2.4. Membrane Morphology and Characterization

The morphology of nanofibrous membranes was observed using a field emission scanning electron microscope (SEM; SUPRA 55 SAPPHIRE, Carl Zeiss AG, Wurttemberg, Germany). The pore size was measured with a capillary flow porometer (CFP-1500AE, PMI Porometer, Ithaca, NY, USA). The water contact angle (WCA) was measured at 25 °C using a contact angle goniometer (SK-CKb, Fengqing Instrument Co., Ltd., Shanghai, China) to analyze the hydrophilicity and hydrophobicity of nanofibrous membranes. Water contact angle was measured using the sessile drop method. For each test, a 3 μL droplet of deionized water was carefully dispensed onto the membrane surface. To ensure data reliability, ten parallel measurements were performed at different positions on each sample, and results are expressed as the mean ± standard deviation (SD). All tests were conducted under controlled conditions: room temperature (25 ± 2 °C) and relative humidity of 50 ± 3%. To eliminate the effect of droplet penetration into the porous membrane, contact angle data were collected 10 s after droplet deposition.

The chemical structure of nanofibrous membranes was investigated by X-ray diffraction (XRD; XRD–7000, Shimadzu Corporation, Kyoto, Japan). The distribution of silver nanoparticles in fibrous membranes was analyzed using energy-dispersive X-ray spectroscopy (EDS; SUPRA 55 SAPPHIRE, Carl Zeiss AG, Germany).

### 2.5. Air Filtration Performance Test

The filtration efficiency and pressure drop of the air filter for NaCl aerosol particles with particle sizes ranging from 300 nm to 10 μm were determined at an air flow rate of 32 L/min using a comprehensive air filter performance tester (LZC-K1, Suzhou Huada Co., Ltd., Suzhou, China). Method for preparing NaCl aerosol: First, dissolve NaCl in deionized water to prepare a solution, and then use an aerosol generator (HRF-4B, Suzhou Hongrui Purification Technology Co., Ltd., Suzhou, China) for preparation. The particles passed through the nanofibrous membranes to the downstream particle collector at different air flow rates. All test data including efficiency and pressure drop were taken as the average of 10 measurements. The filtration efficiency and pressure drop were calculated as follows [32]:

(1) $$\eta =1-\frac{\xi_{2}}{\xi_{1}}$$

(2) $$\Delta P=P_{2}-P_{1}$$

where *ξ*_1_ and *ξ*_2_ represent the particle concentrations at the upstream and downstream of the filter, respectively; *P*_2_ is the upstream pressure and *P*_1_ is the downstream pressure. The quality factor (*Q_F_*, a higher *Q_F_* indicates better air filtration performance) was used to reflect the comprehensive air filtration performance, calculated as follows [33]:

(3) $$Q_{F}=\frac{-\ln\left(1-\eta \right)}{\Delta P}$$

where *η* is the filtration efficiency for 0.3 μm NaCl particles, and Δ*P* is the pressure drop.

### 2.6. Antibacterial Performance Test

According to the specification of GB/T 20944.3-2008 [34], the antibaa performance of nanofibrous membranes was determined by the colony counting method. *Escherichia coli* and *Staphylococcus aureus* were used as the test bacteria. The bacteria were activated at 37 °C for 24 h, and the nanofibrous membranes (0.7 g) were disinfected under UV irradiation for 1 h. Then, 100 μL of bacterial suspension (~10^6^ CFU/mL) of each pathogenic bacterium was added to a test tube containing 70 mL of nutrient broth, and the nanofibrous membranes were immersed in the test tube and shaken at 37 °C and 130 rpm for 12 h. The total number of bacteria in the suspension was calculated by serial dilution plate counting [35]. The inhibition rate (Y) was calculated as follows [36]:

(4) $$Growth\;inhibition\;rate\left(\%\right)=\frac{A-B}{A}\times 100$$

where *A* is the number of colonies in the blank group, and *B* is the number of colonies after treatment with nanofibrous membranes (CFU/mL).

All quantitative tests in this study were performed with 10 independent replicates, and data are presented as mean ± standard deviation (mean ± SD).

## 3. Results and Discussion

### 3.1. Solution Properties

To systematically investigate the effects of chelating/reducing agents on solution properties and subsequent fiber morphology, we designed parallel comparative experiments between PVP and MIPA. Pre-experimental hypotheses suggested that both agents can coordinate with Ag^+^ to regulate its reduction rate and distribution, but differ in their effects on solution rheology and electrospinning behavior: as a high-molecular polymer, PVP forms strong coordination bonds with Ag^+^, which may slow Ag^+^ reduction but significantly alter solution rheology, potentially hindering dendritic fiber formation.

**Figure 1:**
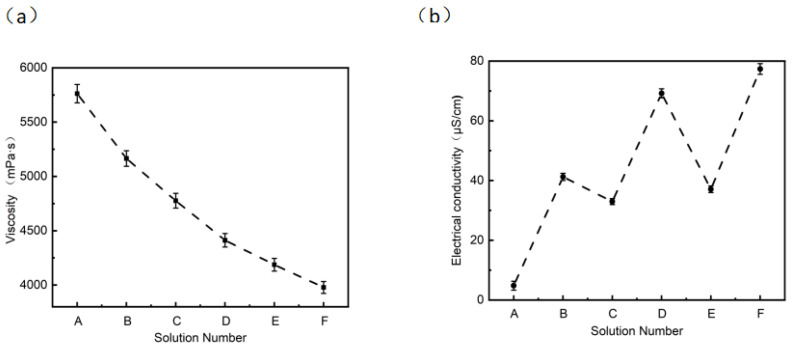
Solution properties of different electrospinning solutions: (**a**) viscosity; (**b**) conductivity; (A) PurePVDF; (B) PVDF/AgNO_3_; (C) PVDF/AgNO_3_/PVP-L; (D) PVDF/AgNO_3_/PVP-H; (E) PVDF/AgNO_3_/MIPA-L; (F) PVDF/AgNO_3_/MIPA-H.

As a small-molecule alcohol amine, MIPA forms weak coordination bonds with Ag^+^ and exhibits mild reducing properties, enabling in situ Ag^+^ reduction during electrospinning. It also has a more pronounced effect on reducing solution viscosity, which is expected to promote jet splitting and dendritic fiber formation. Thus, these experiments aim to identify the optimal chelating–reduction system to achieve synergistic control over silver nanoparticle loading and dendritic fiber structure. In the electrospinning process, solution viscosity and conductivity are two key parameters that directly affect the morphology, structure and final performance of fibers. The diameter, morphology of fibers and the loading effect of silver nanoparticles can be controlled by adjusting the solution viscosity and conductivity, thereby optimizing the filtration and antibacterial performance of membranes [37,38]. Firstly, the viscosities of solutions A, B, C, D, E and F were measured, as shown in Figure 1a. The viscosity of solution A was 5762 mPa·s, and that of solution B was 5165 mPa·s, indicating that the addition of AgNO_3_ reduced the solution viscosity. The viscosities of solutions C, D, E and F were 4776 mPa·s, 4412 mPa·s, 4187 mPa·s and 3978 mPa·s, respectively. It can be seen that the addition of PVP and MIPA also led to a decrease in solution viscosity, with a decrease in solutions E and F. This phenomenon is due to the precipitation of cations that causes the condensation of polymer molecules, leading to the shortening of molecular chains and thus the reduction in solution viscosity [39]. In addition, the viscosity of solution D was lower than that of solution C, and the viscosity of solution F was lower than that of solution E, which indicated that the increase in AgNO_3_ concentration could reduce the solution viscosity in the same solution system. Low-viscosity solutions can more easily form fine jets under the action of an electrostatic field, which is crucial for the formation of dendritic fibers [40]. Dendritic structures usually appear in low-viscosity solutions because the lower viscosity makes the solution more prone to jet splitting under the action of electrostatic force, thus forming fine branched structures.

The conductivities of solutions A, B, C, D, E and F were further measured, as shown in Figure 1b. The conductivity of solution A was 4.82 µs/cm, while that of solution B increased to 41.21 µs/cm. The conductivities of solutions C, D, E and F were 32.96 µs/cm, 69.19 µs/cm, 37.08 µs/cm and 77.33 µs/cm, respectively. It can be seen that after adding PVP and MIPA, the conductivity of the solution decreased significantly. In addition, the conductivity of solution D was higher than that of solution C, and the conductivity of solution F was higher than that of solution E. This indicated that the increase in AgNO_3_ concentration could improve the solution conductivity in the same solution system, because AgNO_3_ can ionize to release silver ions, thus significantly increasing the solution conductivity. Compared with AgNO_3_ solutions with PVP, AgNO_3_ solutions with MIPA had higher conductivity. This is because the carbonyl groups on PVP form coordination bonds with silver ions in the solution, inhibiting the activity of Ag^+^ and reducing the concentration of free Ag^+^ in the solution, while the complexation between MIPA and silver ions is a relatively weak chemical interaction, resulting in a higher concentration of freely mobile Ag^+^ in the solution. In summary, the conductivity of AgNO_3_ solutions with PVP or MIPA decreased, and the conductivity of AgNO_3_ electrospinning solutions with PVP decreased than that with MIPA.

Based on the experimental results and published literature, the optimal viscosity and conductivity ranges for preparing high-performance dendritic fibrous membranes in this study were clarified as follows: Firstly, the optimal viscosity range of the PVDF electrospinning system is 3800–5800 mPa·s. Within this viscosity range, the spinning jet can be fully split under the electrostatic field to form a uniform dendritic structure, while avoiding the formation of defective beaded fibers caused by excessively low viscosity and insufficient jet stretching caused by excessively high viscosity. This range is completely consistent with the classic research conclusion of Zeng et al., who pointed out that solution viscosity directly determines the bending and splitting behavior of the jet during electrospinning, and a moderate viscosity range is the core prerequisite for preparing uniform nanofibers with hierarchical structures [37]. Secondly, the optimal conductivity range of the silver-loaded PVDF electrospinning system is 35–80 μS/cm. Within this conductivity range, the charge-carrying capacity of the solution is significantly improved, which can not only promote the formation of dendritic structures, but also ensure the uniform loading of silver nanoparticles, thus realizing the collaborative optimization of filtration and antibacterial performance of the membranes. This range is fully supported by relevant published studies: Kang et al. pointed out that a solution conductivity of 30–90 μS/cm is the optimal range for the preparation of silver-loaded PVDF nanofiber filtration membranes, which can balance the regulation of fiber morphology and the loading effect of silver nanoparticles [41]; Wu et al. also confirmed that conductivity within this range can achieve high-efficiency filtration performance while maintaining excellent antibacterial activity of the membranes [42].

### 3.2. Morphology Analysis of Fibrous Membranes

The “dendritic fiber structure” is universally defined as a hierarchical tree-like structure with micron-scale main fibers as the matrix, and nanoscale branched fibers grown on the surface of the main fibers. This structure is widely used to improve the specific surface area and filtration performance of nanofibrous membranes, which is completely consistent with both classic and cutting-edge literature reports on dendritic/biomimetic-fractal nanofibers for air filtration [43]. The high-magnification SEM image (Figure S1, Supporting Information) clearly shows the typical hierarchical dendritic structure of the optimal sample in this study.

**Figure 2:**
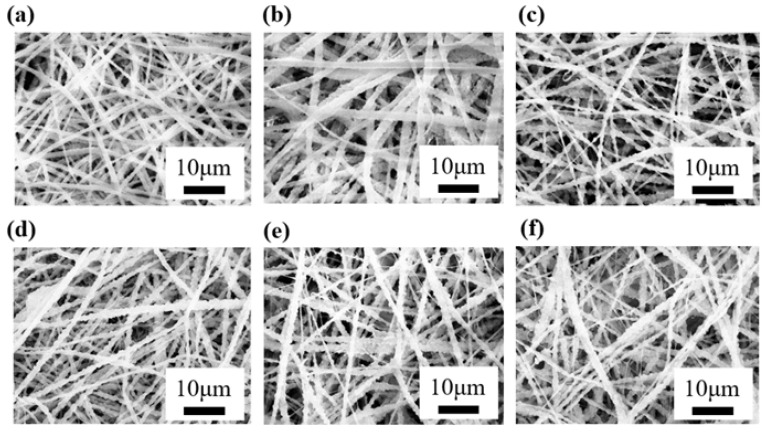
SEM images of nanofibrous membranes prepared from different electrospinning solutions: (**a**) Pure PVDF; (**b**) PVDF/AgNO_3_; (**c**) PVDF/AgNO_3_/PVP-L; (**d**) PVDF/AgNO_3_/PVP-H; (**e**) PVDF/AgNO_3_/MIPA-L; (**f**) PVDF/AgNO_3_/MIPA-H.

The fiber morphology and silver nanoparticle loading of fibrous membranes were observed to explore the effect of silver nanoparticle distribution on the fiber surface on membrane performance, as shown in Figure 2. The results showed that with the addition of silver nanoparticles, dendritic structures appeared on the fiber surface, and the dendritic structures were particularly obvious on the coarser fibers. This indicated that the loading of silver nanoparticles not only affected the fiber diameter but also promoted the formation of dendritic structures, which is consistent with the conclusion that hierarchical bimodal fiber structure can be constructed by adjusting the components of electrospinning solution in related studies [44]. The dendritic fiber structure is mainly characterized by fine branch-like structures distributed between coarser fibers, similar to the branches of a tree, which significantly increases the specific surface area of the fibrous membrane.

Further analysis showed that the formation of dendritic structures was closely related to the viscosity and conductivity of the solution. With the increase in silver concentration from 0% to 0.1%, the fiber diameter increased from about 100 nm to 220 nm, indicating that the addition of silver significantly changed the fiber morphology. The addition of silver nanoparticles significantly improved the solution conductivity and enhanced the charge-carrying capacity of the solution, thus making the jet more prone to splitting under the action of electrostatic force and forming fine dendritic fibers. At the same time, the moderate viscosity of the solution, combined with the complexation of MIPA, can effectively inhibit the migration and micron-scale agglomeration of silver ions, promote the well dispersion of silver nanoparticles on the fiber surface at the micron scale, and enhance the stability of the dendritic structure. The viscosity of sample E was 4187 mPa·s, which was not only in the low viscosity range conducive to the formation of dendritic structure, but also ensured the uniform dispersion of silver nanoparticles through the complexation of MIPA, realizing the collaborative optimization of structure regulation and particle dispersion. Figure 2b–f show the morphologies of silver nanoparticle-loaded fibrous membranes at different silver concentrations. Figure 2b–f show the morphologies of silver nanoparticle-loaded fibrous membranes with different additives. Compared with the pure PVDF membrane (a) and PVDF/AgNO_3_ membrane (b), both PVP-modified (c, d) and MIPA-modified (e, f) samples showed reduced main fiber diameter, induced formation of dendritic branched structures. As clearly demonstrated in the high-magnification SEM images (Figure S2, Supporting Information), significant differences exist between the two additives: compared with PVP-modified samples, MIPA-modified samples exhibit a higher density of dendritic branches, a more uniform fiber diameter distribution, significantly improved fiber surface roughness, and better dispersion of silver nanoparticles on the fiber surface within the resolution of SEM. This difference is attributed to their distinct regulatory effects on the rheological properties of the spinning solution, jet behavior, and silver ion reduction: MIPA has a smaller molecular size, stronger interaction with the solvent, and better complexation with silver ions, which can more significantly reduce the solution viscosity, promote jet splitting, and inhibit silver nanoparticle aggregation, thus forming a denser dendritic network structure with uniformly distributed silver nanoparticles. This structural difference is also the core reason for the better comprehensive performance of MIPA-modified samples. At low silver concentrations (samples B and C), the fiber morphology was relatively uniform with few dendritic structures. With the further increase in silver concentration (samples D, E and F), dendritic structures gradually appeared, and the fiber diameter and the number of dendritic branches increased. Especially in sample E, the dendritic fibers were interleaved between coarse fibers, which further increased the specific surface area of the fibrous membrane and was conducive to enhancing the air filtration efficiency of the membrane. To further quantitatively analyze the fiber size characteristics of the optimal sample E, the fiber diameter distribution was calculated from the SEM images, as shown in Supporting Information Figure S3. The average fiber diameter of sample E is 98.5 nm, with a multi-modal distribution reflecting its hierarchical structure. The proportion of fine dendritic branches (0–80 nm, corresponding to the “fine fibers” discussed) accounts for over 60% of the total fibers, with the highest proportion (37%) in the 40–80 nm range and 23% in the 0–40 nm range. Meanwhile, thick trunk fibers (160–240 nm, corresponding to the “thick fiber” skeleton) make up approximately 30% of the total. These quantitative results confirm the coexistence of fine branched fibers and thick trunk fibers, providing direct support for the synergistic effect of the hierarchical structure on filtration performance (reducing pressure drop while maintaining high efficiency) and antibacterial activity discussed later.

To further characterize the hierarchical structure, the pore size distribution of all samples was analyzed via SEM imaging, as presented in Supporting Information Figure S4. The results verify a dual-scale pore structure: micropores (0.5–2.0 μm) formed by fine dendritic branches enhance filtration efficiency by increasing the probability of particle–fiber collisions, while large through-pores (8–9 μm) formed by thick trunk fibers are in good agreement with the capillary flow porometry (CFP) result of 9.1 μm, which reduces pressure drop by minimizing air flow resistance. A comparison between the two characterization methods clarifies their complementary roles: CFP only measures the effective through-pores that dominate pressure drop, while SEM image analysis captures the full-range pore distribution, including both the main through-pores and the secondary micropores embedded within them. These micropores markedly increase the specific surface area of the fibers, thus boosting particulate interception efficiency. Collectively, the results confirm the synergistic mechanism of “large through-pores reducing pressure drop and micropores improving filtration efficiency”, which explains why sample E achieves the highest filtration efficiency despite possessing the largest through-pore size.

In addition, the dendritic fibers with small diameter and dense distribution effectively reduced the porosity between fibers, thus lowering the particle escape rate. This enabled the dendritic fibrous membrane to capture fine particles more effectively during filtration and improved the membrane filtration efficiency. The formation mechanism of dendritic fiber morphology will be further discussed in Section 3.5 to analyze its influence on fibrous membrane performance. Sample B in the methodological validation group did not form the dendritic fiber structure preset in this study because it did not reduce to silver nanoparticles; therefore, the subsequent structure–activity relationship analysis focuses on the core experimental groups C–F.

### 3.3. Performance Characterization of Nanofibrous Membranes

**Figure 3:**
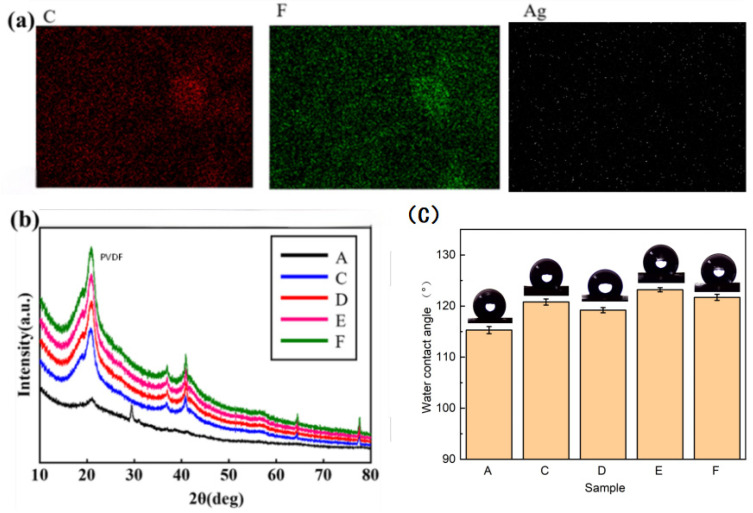
(**a**) EDS image of PVDF/AgNO_3_/MIPA fibrous membrane; (**b**) XRD pattern of silver nanoparticle-loaded fibrous membrane; (**c**) water contact angle of fibrous membranes. Samples A, C, D, E, and F correspond to pure PVDF, PVDF/AgNO_3_/PVP-L, PVDF/AgNO_3_/PVP-H, PVDF/AgNO_3_/MIPA-L, and PVDF/AgNO_3_/MIPA-H, respectively.

Through SEM, EDS, XRD and hydrophobicity tests, the loading effect, reduction state of silver nanoparticles in the fibrous membrane and their influence on membrane performance were systematically revealed. Firstly, the high-magnification SEM image in the Supporting Information Figure S2 shows that spherical nanoparticles with an apparent particle size of 5–20 nm observed by SEM are distributed on the fiber surface of the silver-loaded fibers. The distribution regions of these nanoparticles correspond exactly to the silver elemental distribution in the EDS mapping shown in Figure 3a, which directly confirms the formation of silver nanoparticles. The loading effect of silver nanoparticles in fibrous membranes and their influence on membrane performance were revealed by EDS, XRD and hydrophobicity tests. Firstly, the EDS image of the PVDF/AgNO_3_ fibrous membrane is shown in Figure 3a. Since the main component of nanofibers is PVDF, carbon and fluorine account for the largest proportion. Energy-dispersive X-ray spectroscopy (EDS) mapping results show that in both PVDF/AgNO_3_/PVP composite nanofibers and PVDF/AgNO_3_/MIPA composite nanofibers, silver elements are uniformly distributed on the entire fiber surface without obvious micron-scale elemental segregation. As shown in Table 2, the silver content in the PVP-modified sample is 3.97% by mass and 0.58% by atomic percentage. As shown in Table 3, uniformly distributed silver elements are also detected in the PVDF/AgNO_3_/MIPA composite fibrous membrane, with a mass fraction of 7.21% and an atomic fraction of 1.06%. Combined with the characteristic diffraction peaks of metallic silver in the XRD pattern, it can be confirmed that the complexation system promotes the reduction of silver ions to elemental silver and enables stable attachment to the fiber surface during electrospinning. Unreacted free AgNO_3_ would migrate and aggregate severely during the solvent volatilization stage of electrospinning, which cannot form a uniform Ag element distribution in the fiber, thus ruling out the interference of residual silver salt on the test results. Meanwhile, the significantly higher Ag loading in the MIPA-modified membrane compared with the PVP system directly demonstrated the superior in situ reduction capacity of isopropanolamine: its amino and hydroxyl groups can form stable chelates with Ag^+^ to inhibit ion loss, which is fully supported by the alcohol amine reduction mechanism in reference [45]. The well dispersion of silver species at the micron scale not only optimized the antibacterial performance of the fibrous membrane but also provided a physical basis for the subsequent improvement in air filtration performance. The moderate silver loading can ensure sufficient antibacterial effect on the premise of guaranteeing membrane performance.

Subsequently, the loading state of silver nanoparticles in the fibrous membrane was further verified by XRD analysis, as shown in Figure 3b. In the XRD pattern, the pure PVDF membrane only showed the amorphous diffraction peak characteristic of the PVDF matrix, while in the silver nanoparticle-loaded membrane, the characteristic diffraction peaks of face-centered cubic (fcc) silver were observed, especially at 2θ = 37°, 41°, 61° and 78°, which correspond to the (111), (200), (220) and (311) crystal planes of silver, respectively. This matches perfectly with the standard JCPDS card for metallic silver (No. 04-0783) [46]. The appearance of these characteristic diffraction peaks confirmed the successful loading of silver nanoparticles and indicated that they maintained the fcc structure in the fibrous membrane. As shown in the high-magnification SEM image in Supporting Information Figure S2, within the resolution of SEM and EDS, silver elements show uniform distribution at the micron scale without obvious micron-scale aggregation. This result further proved the good compatibility between silver nanoparticles and the PVDF matrix, and the crystalline structure of the nanoparticles was not affected by the preparation process. Compared with the pure PVDF membrane, the introduction of silver changed the crystal structure of the membrane, thus affecting the physical properties of the membrane, especially playing an important role in improving antibacterial performance and stability. To clarify the physical meaning of this calculation, we explicitly note that the size derived from the Scherrer equation corresponds to the crystallite size of silver nanocrystals, rather than the actual macroscopic particle size of silver nanoparticles. A single silver nanoparticle may consist of multiple aggregated nanocrystallites, leading to distinct differences in both physical definition and numerical value between crystallite size and actual particle size.

To further verify the chemical state of silver and the elemental composition of the fibrous membrane, X-ray photoelectron spectroscopy (XPS) characterization was performed. As shown in Figure S5a, the full-range survey spectrum shows distinct characteristic peaks of C, F and Ag, confirming the successful loading of silver on the PVDF matrix, which is consistent with the EDS elemental analysis results. As shown in Figure S5b, the high-resolution C 1s spectrum exhibits two characteristic peaks at ~284.8 eV and ~290.8 eV, corresponding to the C-C/C-H and -CF_2_- groups of PVDF, respectively, indicating that the chemical structure of the PVDF matrix remains stable after the electrospinning and in situ reduction process. Notably, as shown in Figure S5c the high-resolution Ag 3d spectrum presents two well-resolved characteristic peaks at binding energies of ~368.2 eV (Ag 3d_5_/_2_) and ~374.2 eV (Ag 3d_3_/_2_), with a spin–orbit splitting of 6.0 eV. This feature is fully consistent with the standard binding energy of zero-valent metallic silver (Ag^0^), providing direct spectroscopic evidence for the successful formation of zero-valent silver nanoparticles in the fibrous membrane. Combined with the XRD results, it can be confirmed that the silver nanoparticles prepared via the MIPA-assisted in situ reduction system maintain the crystalline structure of face-centered cubic metallic silver.

Finally, the water contact angle (WCA) test results showed that the hydrophobicity of the fibrous membrane was significantly improved with the loading of silver nanoparticles, as shown in Figure 3c. The WCA of the pure PVDF membrane was 115.3°, while after loading with silver nanoparticles, especially in sample E (PVDF/AgNO_3_/MIPA fibrous membrane), the WCA increased to 123.2°. This indicated that the loading of silver nanoparticles and the formation of dendritic structures were conducive to reducing the contact area between water droplets and the membrane surface, thus enhancing the hydrophobicity of the membrane. Strong hydrophobicity helps to reduce the adhesion of bacteria on the membrane surface because bacteria usually have low adhesion on hydrophobic surfaces, thereby improving the antibacterial performance of the membrane [47].

In addition, the experimental results also showed that the change in WCA was closely related to the silver nanoparticle loading and membrane surface morphology. Compared with PVP, the addition of MIPA promoted the reduction of silver ions, thereby enhancing the loading of silver nanoparticles. However, the WCA of sample D was smaller than that of sample C, and the WCA of sample F was smaller than that of sample E, which indicated that the WCA decreased with the further increase in AgNO_3_ content. This phenomenon may be caused by the change in aggregation state of silver nanoparticles on the fiber surface and the variation in nanofiber surface morphology. Based on the experimental results of this work and well-established academic consensus in the published literature, we clarified the independent roles and synergistic mechanism of isopropanolamine (MIPA), high-voltage electrostatic field, and heat treatment in the in situ formation of silver nanoparticles in this electrospinning system, as detailed below:

First, MIPA acts as the core reducing agent and complexing agent for Ag^+^ reduction in this system, the independent reducing capacity of which has been fully validated in a series of studies on alcohol amine-assisted synthesis of metal nanoparticles. The amino and hydroxyl groups in MIPA molecules have mild reducibility and can serve as electron donors to provide electrons for the reduction of Ag^+^ The reduction of silver ions can be achieved in the alcohol amine–silver salt system at room temperature without external electric field or additional heat treatment, and this reaction pathway has been repeatedly confirmed in relevant studies on alcohol amine–silver salt systems [45]. Meanwhile, MIPA can form weak coordination complexes with Ag^+^, which effectively inhibits the migration and agglomeration of silver ions during solution preparation and electrospinning, and provides sites for the uniform nucleation and growth of silver nanoparticles. This is also the core basis for the favorable dispersion of silver particles in this system.

Second, the high-voltage electrostatic field during electrospinning is the key promoting factor for the in situ reduction of Ag^+^ during jet flight, for which a clear mechanistic consensus has been established in studies on the preparation of metal nanoparticle-loaded polymer fibers via electrospinning with the same system [48]. The X-ray diffraction (XRD) characterization results of this work directly confirmed that the as-spun (unheated) nanofibrous membrane exhibited characteristic diffraction peaks of face-centered cubic metallic silver, which proved that a considerable part of Ag^+^ had been reduced to metallic silver during the electrospinning process only under the action of the electrostatic field. Combined with published research conclusions, the electrostatic field promotes Ag^+^ reduction mainly through two independent pathways: (1) The high-speed stretching of the spinning jet and millisecond-scale rapid solvent volatilization under high voltage sharply increase the local concentrations of Ag^+^ and MIPA in the jet, greatly shortening the induction period of the reduction reaction and accelerating the reduction kinetic process. (2) The electrostatic field can enhance the mobility of free electrons in the system, reduce the activation energy of the Ag^+^ reduction reaction, promote the electron transfer from MIPA to Ag^+^, and directly drive the in situ reduction of silver ions during the jet flight before deposition on the collector.

Third, the subsequent vacuum heat treatment is not the driving force for the initial reduction of Ag^+^, but only plays an optimization role in the reduction degree and crystallinity of silver nanoparticles, for which this functional boundary has been widely verified in relevant studies on polymer-supported metal nanocrystals. The XRD comparison results of this work showed that compared with the unheated as-spun membrane, the heat-treated sample exhibited significantly higher intensity and narrower full width at half maximum (FWHM) of the characteristic diffraction peaks of silver, which is completely consistent with the optimization effect of heat treatment on silver nanocrystals reported in published studies [48]. Specifically, the role of heat treatment is mainly divided into two points: one is to complete the subsequent reduction of residual Ag^+^ in the fibers that is not fully reduced driven by the electrostatic field; the other is to improve the crystallinity of silver nanocrystals, reduce lattice defects, optimize the growth of silver nanocrystals, and further enhance the structural stability of silver nanoparticles through thermal driving. Furthermore, the construction of silver nanoparticle-loaded PVDF composite fibrous membranes via electrospinning has also been demonstrated in other functional systems, such as graphene oxide-modified hybrid membranes for catalytic applications, further verifying the versatility and extensibility of this material system [49].

### 3.4. Air Filtration Performance of Fibrous Membranes

**Figure 4:**
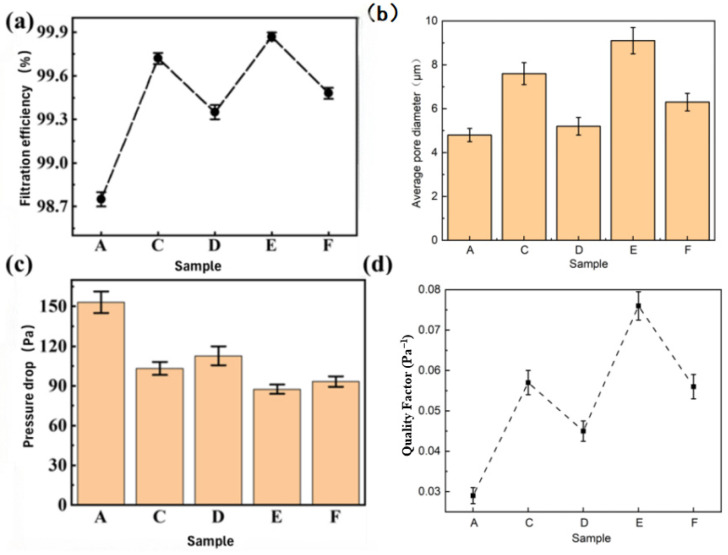
(**a**) Filtration efficiency of different sample membranes; (**b**) average pore diameter of different sample membranes; (**c**) pressure drop of different sample membranes; (**d**) quality factor of different sample membranes. Samples A, C, D, E, and F correspond to pure PVDF, PVDF/AgNO_3_/PVP-L, PVDF/AgNO_3_/PVP-H, PVDF/AgNO_3_/MIPA-L, and PVDF/AgNO_3_/MIPA-H, respectively.

The air filtration performance of PVDF fibrous membranes loaded with different amounts of silver nanoparticles was measured, and the effects of silver concentration, fiber morphology and pore size on the filtration effect were discussed. As shown in Figure 4a, the filtration efficiency of the pure PVDF membrane was 98.75 ± 0.12%, while those of the silver-loaded membranes (C to F) were 99.72% ± 0.08%, 99.35 ± 0.06%, 99.87 ± 0.03% and 99.48 ± 0.05%, respectively. Among them, sample E exhibited the best filtration efficiency. After 14 days of air exposure, as shown in the Supporting Information Figure S6, the filtration efficiencies of samples E and F still remained at 99.51% and 99.24%, showing the chemical stability and performance retention of the membranes within the 14-day test period. Pore size has a direct impact on the filtration performance of fibrous membranes. The pore size test showed that the average pore sizes of samples A, C, D, E and F were 4.8 µm, 7.6 µm, 5.2 µm, 9.1 µm and 6.3 µm, respectively. Compared with the pure PVDF membrane, the loading of silver nanoparticles led to an increase in the membrane pore size, especially for sample E with the largest pore size of 9.1 µm. A moderate silver salt concentration (0.5%) optimized the membrane pore size while maintaining high filtration efficiency and low energy consumption. Excessively high silver concentrations (samples D and F) led to a further increase in pore size, but the corresponding filtration efficiency did not improve further. Therefore, a lower silver concentration helps to maintain a reasonable pore size range, thereby improving filtration efficiency. The filtration efficiency of the membranes against 0.3 μm NaCl particles (the most penetrating particle size, MPPS, for air filtration) was evaluated, and all results are presented as mean ± standard deviation (SD) of 10 independent replicate experiments. One-way analysis of variance (One-way ANOVA) combined with Tukey’s post hoc test was used to assess the statistical significance of differences between groups. The pure PVDF membrane exhibited a filtration efficiency of 98.75 ± 0.12%. For the silver-loaded membranes, the filtration efficiencies were 99.72 ± 0.08% (PVDF/AgNO_3_/PVP-L), 99.35 ± 0.06% (PVDF/AgNO_3_/PVP-H), 99.87 ± 0.03% (PVDF/AgNO_3_/MIPA-L), and 99.48 ± 0.05% (PVDF/AgNO_3_/MIPA-H). The one-way ANOVA results indicated that all silver-loaded samples showed extremely significantly higher filtration efficiency than the pure PVDF control group. Notably, the PVDF/AgNO_3_/MIPA-L membrane achieved the highest filtration efficiency, which was also significantly higher than that of the other silver-loaded samples. These findings confirm that the improvement in filtration efficiency is not due to random experimental error, but rather to the synergistic effect of silver loading modification and the formation of the dendritic fiber structure, which significantly enhances the membrane’s filtration performance.

In terms of pressure drop, the experimental results are shown in Figure 4c. The pressure drop of sample A (pure PVDF membrane) was 153.2 Pa, while those of the silver-loaded membranes were significantly lower. Sample E had the lowest pressure drop of only 87.4 Pa, indicating that the formation of dendritic fiber structures was conducive to reducing the resistance of air flow. The reduction in pressure drop was closely related to the large pore size of sample E, which reduced the resistance of air flow while maintaining high-efficiency particle capture capacity. Ag nanoparticle-decorated PVDF nanofiber/net membranes have also been proven to realize the reduction in air flow resistance while enhancing particulate capture efficiency through the construction of special fiber microstructures, which further verifies the effectiveness of structural optimization strategies for PVDF-based silver-loaded fibrous membranes in improving filtration performance [50]. The QF was further calculated, as shown in Figure 4d. The QF values of samples A, C, D, E and F were 0.029, 0.057, 0.045, 0.076 and 0.056, respectively. Sample E had the highest QF value of 0.076, indicating that this sample achieved the best comprehensive performance with high filtration efficiency and low pressure drop. The improvement in QF value was closely related to the formation of dendritic structures. The presence of dendritic fibers increased the specific surface area of the membrane, enabling the membrane to effectively filter fine particles at low energy consumption. The MIPA-modified sample with 0.05 g low loading exhibited the highest filtration efficiency (99.87%) and the lowest pressure drop (87.4 Pa), with a quality factor (0.076 Pa^−1^) higher than that of the 0.1 g high-loading sample (0.056 Pa^−1^). An excessively high silver nitrate loading would lead to too low solution viscosity, collapse of the dendritic structure, disordered fiber deposition, a significant increase in filtration pressure drop, and thus a decline in overall filtration performance.

From the perspective of overall performance, the silver nanoparticle-loaded fibrous membrane achieved a good balance among filtration efficiency, pressure drop and QF. Sample E showed the best balance in high efficiency, low pressure drop and high QF, which proved the significant improvement in the synergistic effect of silver nanoparticle loading and dendritic fiber structure on fibrous membrane performance. The dendritic structure not only increased the specific surface area of the membrane but also reduced the air flow resistance by improving the pore size distribution and optimizing the interfiber structure, thus further enhancing the filtration effect of the membrane. In summary, the silver nanoparticle-loaded fibrous membrane has good air filtration performance, especially sample E with a filtration efficiency of 99.87%, the minimum pressure drop and the highest quality factor. These results indicate that by adjusting the silver nanoparticle loading and pore size distribution, the comprehensive performance of the membrane can be improved with high filtration efficiency and low pressure drop guaranteed. While previous studies have suggested a potential correlation between PVDF β-phase content and surface electrostatic adsorption, no quantitative analysis of β-phase content was performed in this work, and its direct relationship with filtration performance cannot be confirmed. The core enhancement in filtration efficiency observed here is attributed to the optimized dendritic hierarchical structure, which provides enhanced physical interception and air flow regulation. The potential influence of PVDF crystal phases remains to be systematically investigated in future work.

To further clarify the practical application potential of the membranes prepared in this study, the sample E with the best overall performance was benchmarked against mainstream commercial polypropylene (PP) melt-blown filter media currently used in air filtration and respiratory protection. Existing similar studies have widely reported the typical performance parameters of commercial melt-blown filter materials: under a testing face velocity of 5.3 cm/s, which is exactly the same as in this study, KN95-grade commercial PP melt-blown filter media typically achieve a filtration efficiency of 95–99% for 0.3 μm NaCl particles, with a corresponding pressure drop of 100–200 Pa. The quality factor (QF), the core indicator representing comprehensive filtration performance, is only 0.01–0.03 Pa^−1^ [51]. At the same time, commercial melt-blown filter media generally lack inherent antibacterial properties, and prolonged use can easily lead to bacterial growth, causing secondary contamination.

### 3.5. Air Filtration Mechanism

The air filtration mechanisms mainly include five types: diffusion effect, inertial impaction, interception effect, gravitational sedimentation and electrostatic effect. The diffusion effect and electrostatic effect have better capture effects on particulates smaller than 0.3 µm, while 0.3 µm particulates are the most difficult to remove [52]. Therefore, the diffusion effect and electrostatic effect play an important role in the high-efficiency capture of fine particulates.

In this study, the prepared silver nanoparticle-loaded PVDF fibrous membrane had a small fiber diameter, and the intrinsic electronic effect of silver nanoparticles significantly enhanced the diffusion and electrostatic effects. Silver nanoparticles promoted the enhancement of the electrostatic effect by providing electronic loading, which is crucial for capturing particulates smaller than 0.3 µm. In this study, the improvement effect of silver nanoparticles on the electrostatic adsorption performance of nanofiber membranes is completely consistent with the classic research conclusions in the field of electrospun silver nanoparticle-loaded antibacterial filter membranes. The relevant literature has directly confirmed this conclusion through Zeta potential testing, fully demonstrating that silver nanoparticles can significantly increase the surface charge and charge density of polymer nanofibers, thereby enhancing their electrostatic capture capability [53].

In addition, the introduction of the dendritic fiber structure further optimized the diffusion effect. The dendritic structure increased the collision probability between particulates and fibers, improving the particulate capture efficiency.

On the other hand, the influence of the fibrous membrane on air flow is also an important factor affecting air filtration performance. With the decrease in fiber diameter, the slip effect gradually appears. The fiber diameter distribution chart of sample E is based on the measured fiber diameters, for the quantitative calculation of the Knudsen number (*K_n_*) and the determination of flow regime. The calculation formula is as follows:

(5) $$K_{n}=\frac{\lambda }{L}$$

In the formula: *K_n_* is the Knudsen number; λ is the mean free path of air molecules at standard temperature and pressure (25 °C, 101.325 kPa), taking the industry-standard value of 67 nm; *L* is the fiber diameter, measured in nm.

Quantitative analysis (Table S1) reveals that all fibers in sample E are in the transitional flow regime (0.1 < *K_n_* < 10), where the gas slip effect is non-negligible for air flow resistance. Specifically, the finest dendritic branches 0–80 nm, accounting for ~60% of total fibers, possess *K_n_* values greater than 1, inducing a dominant strong gas slip effect that serves as the primary contributor to the membrane’s low pressure drop. In contrast, the thicker fibers, including 80–120 nm and 200–240 nm fractions, which represent ~40% of total fibers, have *K_n_* values between 0.3 and 0.7 (*K_n_* < 1). Although their gas slip effect is weaker than that of the finer fibers, their larger diameters construct more interconnected and large-sized pore channels for air flow, which further reduces the overall flow resistance of the membrane.

When the fiber diameter is less than 1 µm, the air flow can bypass the fibers, reducing energy loss and thus providing a basis for low-resistance filtration. When the fiber diameter is reduced to below 60 nm, the slip effect is further enhanced, leading to a further reduction in resistance. Especially when the fiber diameter is reduced to below 13 nm, the resistance is almost negligible. However, as an aggregate of fibers, the overall packing effect of the fibrous membrane will cause more obstacles to the air flow passing through the membrane, thus increasing the resistance. The introduction of the dendritic structure effectively balanced this problem. The fine branch fibers enhanced the slip effect, while the coarser main fibers provided more bypass space for the air flow, enabling the air flow to pass through more smoothly, thus maximizing the advantages of the slip effect and reducing the resistance.

**Figure 5:**
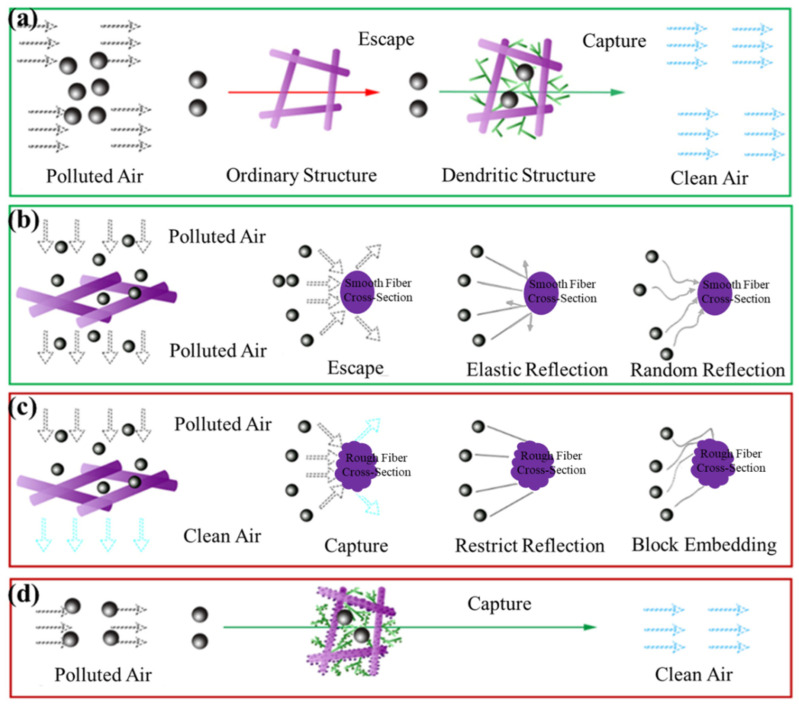
(**a**) Schematic diagram of high-efficiency filtration by dendritic structure; schematic diagrams of nanofiber filtration mechanisms: (**b**) smooth fiber; (**c**) rough fiber; (**d**) schematic diagram of air filtration by the prepared rough dendritic silver nanoparticle-loaded fibrous membrane.

The improvement in the dendritic structure on filtering performance is mainly reflected in the enhancement of the interception effect. Based on the fiber diameter data measured in this study, the classic single-fiber filtration model by Lee & Liu, commonly used in the field of air filtration, was applied to quantitatively separate the contribution proportions of various capture mechanisms for 0.3 μm NaCl particles [54]. The results are shown in Table S2. The improvement in air filtration performance by the dendritic structure is mainly reflected in the enhancement of the particulate interception effect. The diffusion effect mainly affects the capture of small particulates, while the dendritic structure significantly enhances the particulate interception efficiency by increasing the collision probability between particulates and fibers. The multi-level space between the fine branches of the dendritic structure and the fibers makes the particulates undergo more collisions and rebounds during the filtration process, thereby reducing the particulate escape and enhancing the capture capacity of the membrane. The specific surface area of the membrane is significantly increased through the dendritic structure design, further improving the particulate interception capacity, as shown in Figure 5a.

In addition, the influence of fiber surface roughness on filtration performance cannot be ignored; the roughness of the fiber surface can significantly enhance the air filtration performance of the fiber membrane [55]. As shown in Figure 5b,c, the smooth fiber surface has a small contact area with particulates, and particulates are prone to elastic reflection, leading to their escape from the fiber surface and failure to be effectively captured. In contrast, the rough fiber surface provides more contact points. When particulates collide with the fiber surface, they not only reflect at multiple angles but may also be embedded in the surface depressions, thus improving the filtration efficiency. The formation of rough surfaces involves various mechanisms, including micro-surface structure changes caused by solvent volatilization, or inherent rough structures formed during film formation due to the combination of different substances in the solution. The presence of a rough surface greatly increases the contact area between fibers and particulates, thereby improving the filtration performance. The synergistic effect of the dendritic structure and rough surface makes the silver nanoparticle-loaded fibrous membrane show significant advantages in air filtration. As shown in Figure 5d, the dendritic structure not only effectively improves the filtration efficiency but also optimizes the pore structure and reduces the air flow resistance, further enhancing the overall filtration performance of the membrane. Silver nanoparticles not only improve the filtration efficiency by enhancing the electrostatic effect but also extend the service life of the membrane by providing antibacterial performance. Therefore, the synergistic optimization of dendritic structure and rough surface is the core reason for the silver nanoparticle-loaded fibrous membrane to achieve high efficiency and low-resistance air filtration. This apparent contradiction can be explained by the hierarchical pore structure of the membrane, which deviates from the core premise of traditional filtration theory. The conventional rule that “smaller pore size leads to higher efficiency” applies only to membranes with a single-level pore structure, smooth fibers, and a single filtration mechanism. In contrast, the dendritic fibrous membrane in this work has a multiscale hierarchical structure, and its filtration performance arises from the synergistic action of multiple mechanisms, rather than being determined by a single pore size parameter.

To further elucidate this, we combined SEM and CFP characterization to analyze the hierarchical pore structure of sample E. The large through-pores (average 9.1 μm) formed by thick trunk fibers, which are the main signal detected by CFP, provide low-resistance pathways for air flow, reducing pressure drop. Meanwhile, a large number of fine pores (<2 μm) formed by dendritic fine fibers significantly increase specific surface area of the fiber, enhancing particle interception, diffusion, and electrostatic adsorption—key mechanisms for achieving high filtration efficiency. This structural feature directly demonstrates the enhancement effect of the dendritic structure on the membrane’s particle capture capacity.

### 3.6. Antibacterial Performance of Fibrous Membranes

**Figure 6:**
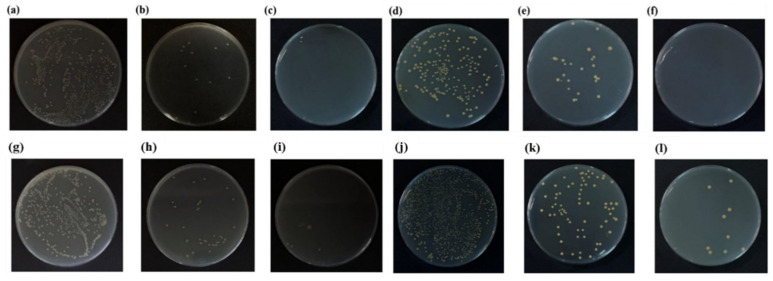
Antibacterial experiments against *Escherichia coli*: (**a**) pure PVDF; (**b**) PVDF/AgNO_3_/PVP-L; (**c**) PVDF/AgNO_3_/MIPA-L. Antibacterial experiments against *Staphylococcus aureus*: (**d**) pure PVDF; (**e**) PVDF/AgNO_3_/PVP-L; (**f**) PVDF/AgNO_3_/MIPA-L. Antibacterial experiments against *Escherichia coli* after two weeks: (**g**) pure PVDF; (**h**) PVDF/AgNO_3_/PVP-L; (**i**) PVDF/AgNO_3_/MIPA-L. Antibacterial experiments against *Staphylococcus aureus* after two weeks: (**j**) pure PVDF; (**k**) PVDF/AgNO_3_/PVP-L; (**l**) PVDF/AgNO_3_/MIPA-L. Antibacterial performance of the nanofibrous membranes against *Escherichia coli* (ATCC 25922) and *Staphylococcus aureus* (ATCC 25923). The antibacterial test was performed in accordance with GB/T 20944.3-2008 standard. All experiments were conducted in 10 independent replicates, and images were captured under identical conditions (brightness, contrast, exposure time) to ensure consistent comparison.

To evaluate the antibacterial effect of silver nanoparticle-loaded PVDF fibrous membranes, *Escherichia coli* and *Staphylococcus aureus* were used as test strains to conduct antibacterial performance tests on fibrous membranes with different silver concentrations. The experimental results are shown in Figure 6. All antibacterial tests were conducted with 10 independent parallel experiments, and results are presented as mean ± standard deviation (SD). Antibacterial stability within the 14-day test cycle was evaluated, with error bars added to all data points to reflect test variability. One-way ANOVA combined with Tukey’s post hoc test was performed to assess significant differences in antibacterial performance between groups. The results show that sample E exhibited significantly higher inhibition rates against both *E. coli* and *S. aureus* than sample C throughout the 14-day test, demonstrating the superior long-term antibacterial efficacy of the MIPA-modified system.

The silver nanoparticle-loaded fibrous membrane exhibited significant inhibitory effects on both common pathogenic bacteria. In the antibacterial experiments of *Escherichia coli* and *Staphylococcus aureus*, sample E had inhibition rates of 99.9% and 99.8% against the two bacteria, respectively, while the pure PVDF membrane had almost no inhibitory effect on them. To further verify the durability of antibacterial performance, exposure tests were also carried out on the silver nanoparticle-loaded membranes. Samples C and E were exposed to the air for two weeks, as shown in the Supporting Information Figure S7, and the results showed that their antibacterial performance still remained at a high level. After 14 days of exposure, sample E had inhibition rates of 98.1% and 97.2% against *Escherichia coli* and *Staphylococcus aureus*, respectively, and sample C had inhibition rates of 91.8% and 93.1%, respectively. These results indicated that the silver nanoparticle-loaded fibrous membrane could maintain high antibacterial activity within the 14-day test period, and its antibacterial effect was stable without significant attenuation over the 14-day test cycle.

The antibacterial performance of silver nanoparticles mainly comes from their loading on the membrane surface [56]. When bacteria come into contact with silver nanoparticles, silver ions can be released and react with the bacterial cell membrane, leading to the rupture of the cell membrane, leakage of intracellular contents and cell death. The antibacterial mechanism of silver nanoparticles is mainly realized through pathways such as destroying the bacterial cell membrane and inhibiting cell respiration and reproduction [57]. In addition, the dendritic structure and rough surface of the silver nanoparticle-loaded fibrous membrane also play a promoting role in antibacterial performance. The rough surface and dendritic structure increase the contact area between the membrane and bacteria, improve the release efficiency of silver ions, and further enhance the antibacterial effect. The dendritic fibers increase the contact probability between bacteria and the membrane by increasing the contact area, making more bacteria exposed to the action of silver nanoparticles and increasing the chance of antibacterial reactions.

The core innovations and scientific contributions of this work, which go far beyond simple process parameter optimization, are summarized as follows:

A one-step MIPA-assisted in situ reduction system was developed to construct silver nanoparticle-loaded PVDF dendritic fibrous membranes, enabling synergistic control over fiber morphology, silver loading distribution, and hierarchical pore structure, a distinct approach from conventional preparation routes.

The mechanism by which the dendritic hierarchical structure mitigates the “filtration efficiency-pressure drop” trade-off was elucidated: thick trunk fibers form large through-pores to reduce air flow resistance, while fine dendritic branches enhance particle capture efficiency, achieving simultaneous high antibacterial activity, ultra-high filtration efficiency, and low pressure drop at low silver loading.

This work provides a novel structural design strategy and facile fabrication route for PVDF-based nanofibrous membranes with combined high-efficiency antibacterial and low-resistance filtration performance, complementing current understanding of how dendritic fiber structures modulate filtration behavior.

## 4. Conclusions

Aiming at the current problem that it is difficult to balance antibacterial and filtration performance of metal nanoparticle-loaded fibrous membranes, this study proposed a new solution. Silver nanoparticle-loaded dendritic fibrous membranes were prepared by the electrospinning process, and the dual optimization of antibacterial and filtration performance was achieved by adjusting the silver loading amount and fiber morphology. The prepared silver nanoparticle-loaded PVDF dendritic fibrous membrane exhibited remarkable air filtration performance with a filtration efficiency of 99.87% for 0.3 µm particulate matter, a pressure drop of 87.4 Pa and a QF of 0.076 Pa^−1^. In addition, the membrane had inhibition rates of 99.9% and 99.8% against Escherichia coli and *Staphylococcus aureus*, respectively, and the inhibition rates still reached 98.1% and 97.2% after 14 days of membrane exposure, showing good antibacterial stability within the 14-day test period. A comparison with silver-loaded PVDF filter membranes reported in the recent literature confirms the differentiated advantages of this work. The dendritic hierarchical membrane achieves a higher quality factor (QF) than most previously reported materials, demonstrating superior capability in balancing high filtration efficiency and low pressure drop. Meanwhile, its 14-day antibacterial rates against *Escherichia coli* and *Staphylococcus aureus* also outperform many comparable silver-loaded PVDF membranes, highlighting the effectiveness of the MIPA modification strategy. This study provides a new insight into resolving the trade-off between air filtration and antibacterial performance of metal nanoparticle-loaded fibrous membranes and offers an important reference for applications in related fields.

## Figures and Tables

**Table 1:** Formulation of electrospinning solutions for different membrane samples.

Sample No.	PVDF (g)	AgNO_3_ (g)	PVP (g)	MIPA (g)	DMF (g)	AC (g)
A	0.9	-	-	-	7.28	2.82
B	0.9	0.05	-	-	7.28	2.82
C	0.9	0.05	0.14	-	7.28	2.82
D	0.9	0.1	0.14	-	7.28	2.82
E	0.9	0.05	-	0.14	7.28	2.82
F	0.9	0.1	-	0.14	7.28	2.82

**Table 2:** Element content distribution of PVDF/AgNO_3_/PVP fibrous membrane.

Element	Weight Percentage (%)	Atomic Percentage (%)
C	50.78	63.14
F	45.25	36.28
Ag	3.97	0.58

**Table 3:** Element content distribution of PVDF/AgNO_3_/isopropanolamine fibrous membrane.

Element	Weight Percentage (%)	Atomic Percentage (%)
C	49.19	57.75
F	43.6	41.19
Ag	7.21	1.06

## Data Availability

The original contributions presented in this study are included in the article/Supplementary Materials. Further inquiries can be directed to the corresponding author.

## Supplementary Materials

The following supporting information can be downloaded at https://www.mdpi.com/article/10.3390/mi17050614/s1, Figure S1: Dendritic fibers and bead SEM comparison image. Figure S2: High-magnification SEM images of nanofibrous membranes. Figure S3: Fiber Diameter Distribution Chart of AgNO_3_/MIPA-L. Figure S4: Pore size distribution under SEM. Figure S5: X-ray photoelectron spectroscopy (XPS) characterization of the PVDF/AgNO_3_/MIPA-L composite membrane. Figure S6: Filtration performance after two weeks of exposure. Figure S7: Antibacterial performance after two weeks of exposure. Table S1: Fiber diameter distribution of sample E. Table S2: Contribution of each capture mechanism of fibers to 0.3 μm NaCl particles.

## Abbreviations

The following abbreviations are used in this manuscript:

AC	Acetone
Cu	Copper
CuO	Copper(II) oxide
Cu_2_O	Copper(I) oxide
AgNO_3_	Silver nitrate
CFU	Colony-Forming Unit
CFP	Capillary Flow Porometer
DMF	N,N-Dimethylformamide
EDS	Energy-Dispersive X-ray Spectroscopy
MIPA	Isopropanolamine
ΔP	Pressure Drop
PVDF	Polyvinylidene fluoride
PVP	Polyvinylpyrrolidone
Q_F_	Quality Factor
ROS	Reactive Oxygen Species
SEM	Scanning Electron Microscope
WCA	Water Contact Angle
XRD	X-ray diffractometer/X-ray diffraction
NaCl	Sodium chloride
