# Supplementary material for: Preparation and Optimization of Silver Nanoparticle-Loaded Dendritic Fibrous Membranes for High-Efficiency Antibacterial Activity and Air Filtration
Source: Micromachines (Basel). 2026 May 16;17(5):614. doi: 10.3390/mi17050614 (PMC13209917; doi:10.3390/mi17050614)
Supplement: Supplementary file 1 [file micromachines-17-00614-s001.zip › micromachines-4251840-supplementary.pdf]

Supporting Information

This document includes 7 supplementary figures (Figure S1 to Figure S7) and 2 supplementary tables (Table S1 to Table S2).

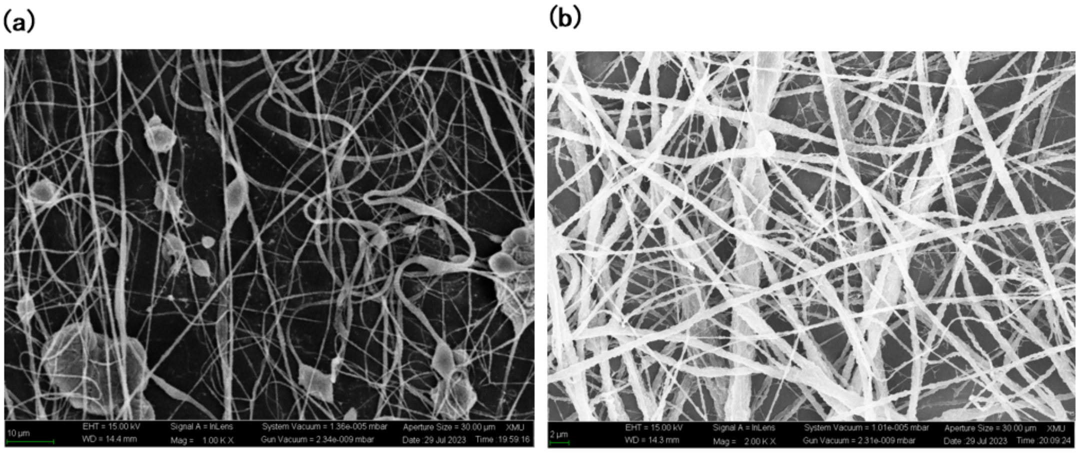

Figure S1. Dendritic fibers and bead SEM comparison image; (a): PVDF/$AgNO_3$,Beaded fiber SEM image; (b): PVDF/$AgNO_3$/MIPA-L,Dendritic fiber SEM image

c

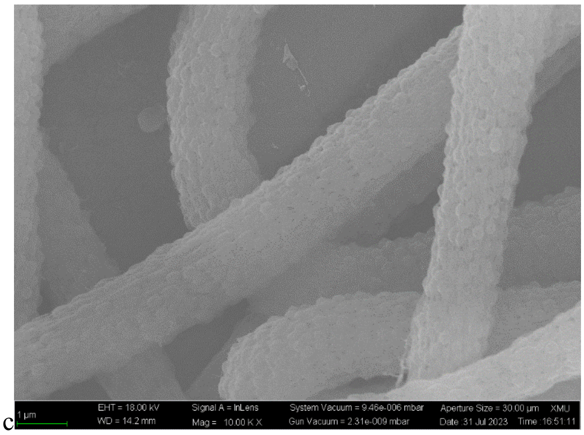

Figure S2. High-magnification SEM images of nanofibrous membranes

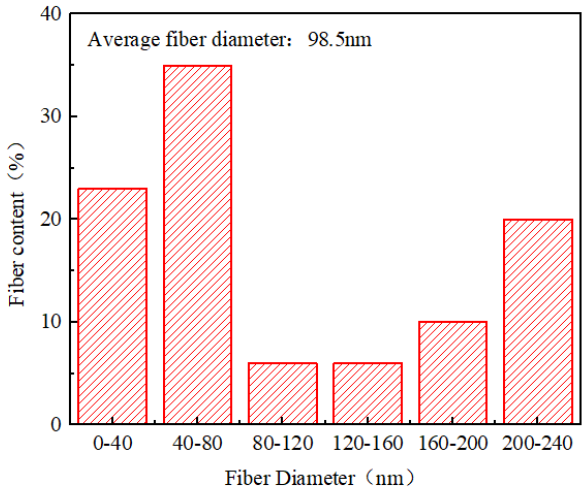

Figure S3. Fiber Diameter Distribution Chart of $AgNO_3$/MIPA-L

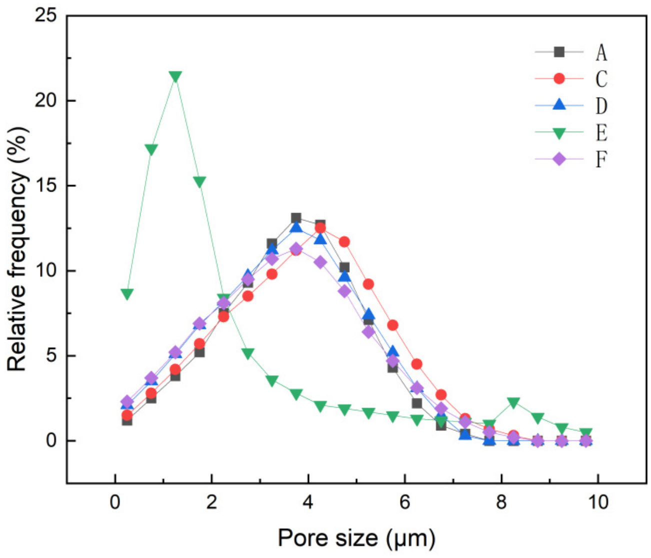

Figure S4. Pore size distribution under SEM

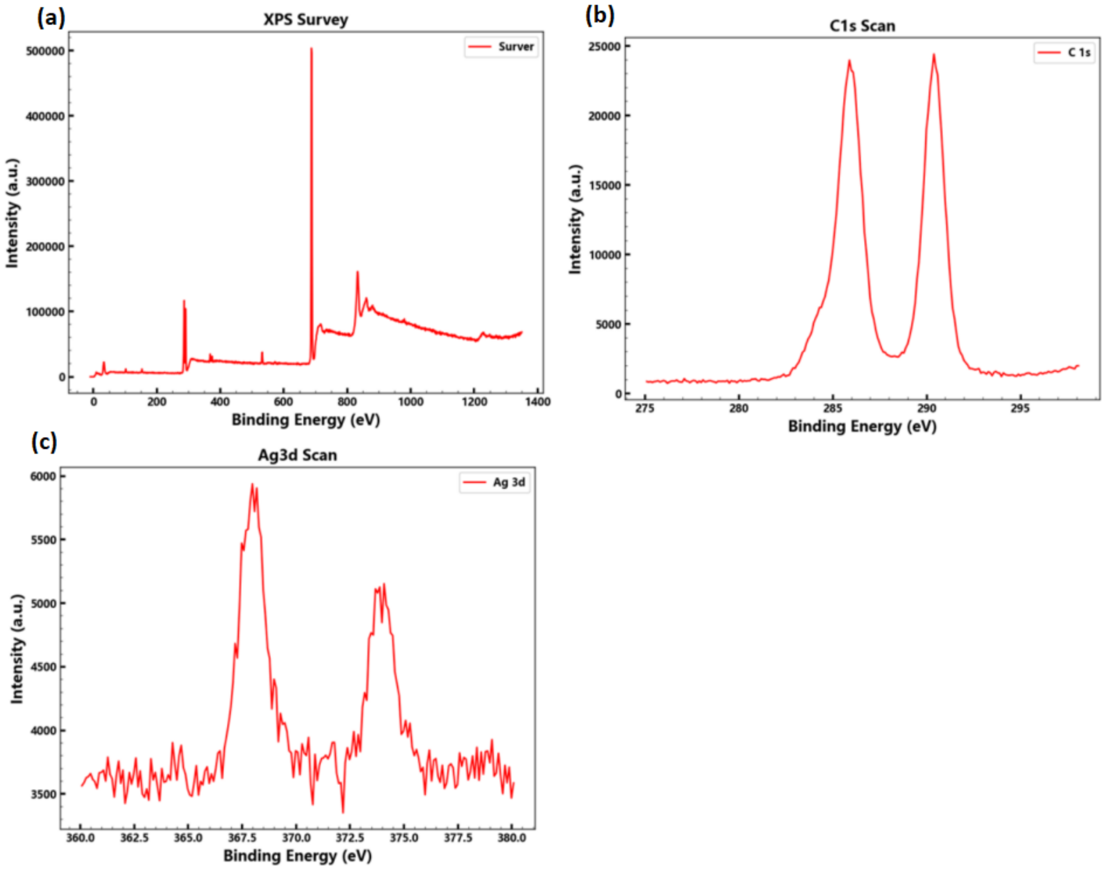

Figure S5. X-ray photoelectron spectroscopy (XPS) characterization of the PVDF/$AgNO_3$/MIPA-L composite membrane: (a) full survey spectrum, (b) high-resolution C 1s spectrum, (c) high-resolution Ag 3d spectrum.

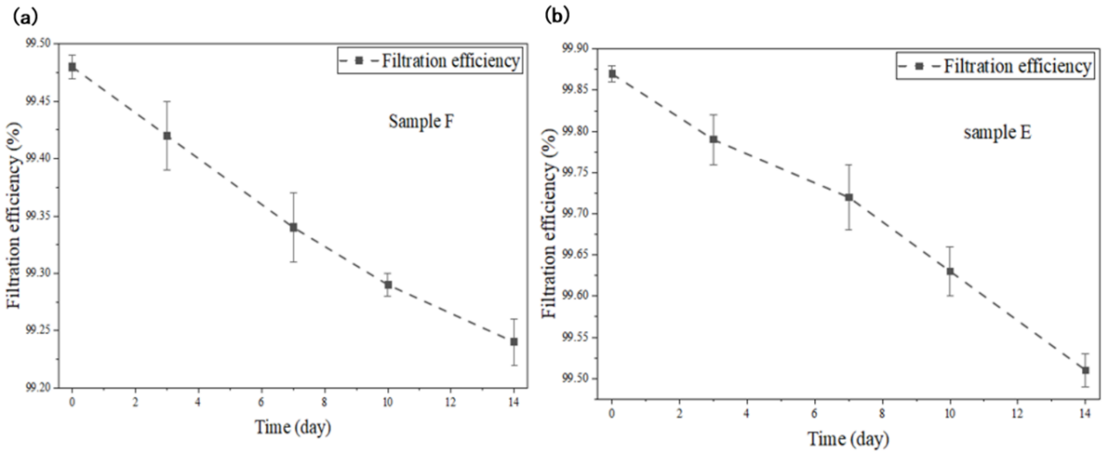

Figure S6. Filtration performance after two weeks of exposure;(a):$AgNO_3$/MIPA-H;(b):$AgNO_3$/MIPA-L

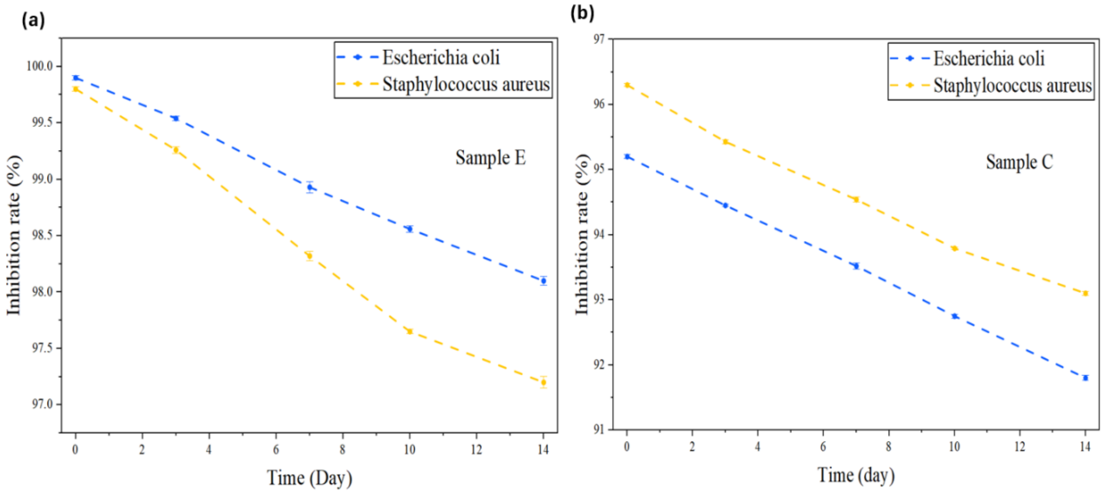

Figure S7. Antibacterial performance after two weeks of exposure; (a) $AgNO_3$/MIPA-L; (b) $AgNO_3$/PVP-L

Table S1. Fiber Diameter Distribution of Sample E

Fiber diameter range	Knudsen number Kn	Flow regime interval
0-40 nm	3.350	transitional flow
40-80 nm	1.117	transitional flow
80-120 nm	0.670	transitional flow
120-160 nm	0.479	transitional flow
160-200 nm	0.372	transitional flow
200-240 nm	0.305	transitional flow

Table S2. Contribution of each capture mechanism of fibers to 0.3 μm NaCl particles

Capture mechanism	Contribution proportion of primary fibers	Contribution proportion of dendritic fine fibers
Inertial impaction	0.12%	0.38%
Interception effect	7.25%	13.12%
Diffusion effect	21.63%	31.50%
Electrostatic adsorption	71.00%	55.00%
